# Metabolic and Molecular Mechanisms of Macrophage Polarisation and Adipose Tissue Insulin Resistance

**DOI:** 10.3390/ijms21165731

**Published:** 2020-08-10

**Authors:** Lucie Orliaguet, Tina Ejlalmanesh, Fawaz Alzaid

**Affiliations:** Cordeliers Research Centre, INSERM, IMMEDIAB Laboratory, Sorbonne Université, Université de Paris, F-75006 Paris, France; lucie.orliaguet@gmail.com (L.O.); tina.ejlalmanesh@crc.jussieu.fr (T.E.)

**Keywords:** adipose tissue, inflammation, insulin resistance, immunometabolism, macrophages, type-2 diabetes, T2D

## Abstract

Inflammation plays a key role in the development and progression of type-2 diabetes (T2D), a disease characterised by peripheral insulin resistance and systemic glucolipotoxicity. Visceral adipose tissue (AT) is the main source of inflammation early in the disease course. Macrophages are innate immune cells that populate all peripheral tissues, including AT. Dysregulated AT macrophage (ATM) responses to microenvironmental changes are at the root of aberrant inflammation and development of insulin resistance, locally and systemically. The inflammatory activation of macrophages is regulated at multiple levels: cell surface receptor stimulation, intracellular signalling, transcriptional and metabolic levels. This review will cover the main mechanisms involved in AT inflammation and insulin resistance in T2D. First, we will describe the physiological and pathological changes in AT that lead to inflammation and insulin resistance. We will next focus on the transcriptional and metabolic mechanisms described that lead to the activation of ATMs. We will discuss more novel metabolic mechanisms that influence macrophage polarisation in other disease or tissue contexts that may be relevant to future work in insulin resistance and T2D.

## 1. Introduction: Physiology and Pathology of Adipose Tissue

The physiological role of adipose tissue (AT) is long-term energy storage in the form of fat, and depending on the AT depot, this fat also provides protection and insulation. AT is also an endocrine organ and responds to endocrine signalling to regulate appetite and to control systemic lipid homeostasis. Persistent overnutrition over time leads to adipose tissue expansion, phenotypic alterations and changes in sensitivity to hormone signalling.

AT is composed of two main fractions: the adipocyte fraction and the stromal vascular fraction (SVF). Adipocytes form the main component regulating energy stores and systemic lipid homeostasis. The SVF fraction is heterogenous in composition and changes over time in response to altered metabolic needs. The SVF is composed of mesenchymal progenitor/stem cells, preadipocytes, fibroblasts, endothelial cells, and immune cells including macrophages [1]. Macrophages represent one of the most dynamic cells in this fraction of adipose tissue and are key actors of inflammation and the development of insulin resistance.

Under physiological conditions, AT macrophages (ATMs) represent approximately 2% of the cells in AT. Throughout the development of obesity, insulin resistance and type-2 diabetes (T2D), ATMs increase in number to represent up to 50% of cells in AT [1]. The increase in ATM numbers is due to proliferation and the recruitment of circulating monocytes that differentiate in situ into macrophages. The increase in ATM number is accompanied by an altered phenotype. Whilst macrophages in the lean and metabolically healthy state are alternatively activated (M2-like or anti-inflammatory phenotype), macrophages in the insulin resistant state are classically or metabolically activated (M1-like, pro-inflammatory or intermediate phenotypes) [2,3,4]. The increased number and change in polarisation are accompanied by an increase in pro-inflammatory cytokines, in the microenvironment and systemically.

This review will address the established inflammatory mechanisms implicated in adipose insulin resistance. Particular attention is drawn to the transcriptional-metabolic cross-talk in macrophages that optimises or mitigates AT adaptation to systemic and micro-environmental dysmetabolism. However, within the scope of inflammation and metabolic diseases, several questions remain unanswered. Namely the factors that initiate inflammation and ATM activation, such as hyperlipidaemia, hyperglycaemia, adipocyte death or release of exosomes. Similarly, the tissue specificity of macrophages remains to be studied in detail. Whilst progress has been made in defining cell lineages and differentiation trajectories, single-cell analyses and high density in situ analytical methods have only started to unravel the metabolic and transcriptional specificities of ATM subsets relative to other macrophages (monocyte-derived, peritoneal, Kupffer or red pulp). In this light, the current review brings together the established transcriptional and metabolic mechanisms that dictate macrophage effector functions and inflammatory polarisation in contexts relevant to insulin resistance and T2D.

## 2. Pathways Involved in Adipose Tissue Inflammation and Insulin Resistance

Studies in the 1990s drew the earliest links between inflammation and adipose tissue insulin resistance. Hotamisligil et al. and Uysal et al. demonstrated that the pro-inflammatory cytokine tumour necrosis factor (TNF) was highly expressed in AT of obese rodents and that this cytokine was causally linked to insulin resistance by direct interference with insulin receptor signalling [5,6,7].

Finding the precise triggers of this inflammatory response is an area of active research. However, expanding AT provides a number of potential cues that can influence macrophage polarisation, for example, adipocyte death or senescence, hypoxia, increased lipolysis or mechanical stress. Recent research has even implicated insulin resistance itself in the initiation of inflammation [8]. Indeed, it is important to note that in long-term diet-induced obesity, two phases of insulin resistance exist; an early phase that is independent of inflammation and an inflammation-dependent late phase [9].

More and more studies have implicated extracellular vesicles as important communication molecules between adipocytes and macrophages in the initiation or control of inflammation. These vesicles are exosomes released from adipocytes and from adipose tissue-derived mesenchymal stem cells and can influence insulin signalling, inflammation, and angiogenesis. Indeed, adipose exosomes carry hormones such as adiponectin, inflammatory adipokines (tumour necrosis factor, TNF; macrophage colony-stimulating factor, MCSF), fatty acid transporters (AFBP4) and miRNAs. Exosome composition varies between metabolic states and the different components have been predicted to regulate transforming growth factor (TGF)-beta and wnt/beta-catenin pathways [10].

## 3. Overview of Insulin Signalling, Resistance and the Onset of Type-2 Diabetes

Insulin exerts its effects through binding to its cell surface receptor, which undergoes autophosphorylation. Phosphorylation of three tyrosine residues is necessary for amplification of kinase activity that recruits insulin receptor substrate (IRS) proteins important for the signalling pathway. Downstream of these events, phosphatidylinositol-3-kinase (PI3K) and mitogen-activated protein kinase (MAP-kinase) mediate the metabolic and mitogenic actions of insulin in the cell [11].

Insulin resistance is a result of failure of one or more of these mechanisms, resulting in decreased glucose uptake, glycogen synthesis (mainly by the liver) and increased lipolysis in AT. Resulting hyperglycaemia and dyslipidaemia potentiate insulin secretion from the pancreas. If sustained, this vicious cycle results in beta cell failure, frank T2D and increased risk of complications and comorbidities. Chronic inflammation has been consistently implicated at every step of these changes [12,13,14]. Moreover, numerous studies have demonstrated that blunting the inflammatory response, mitigates metabolic decline, preserves glycaemic homeostasis or even reverses insulin resistance [14,15,16].

## 4. Molecular Mechanisms of Inflammation and Adipose Insulin Resistance

**Figure 1:**
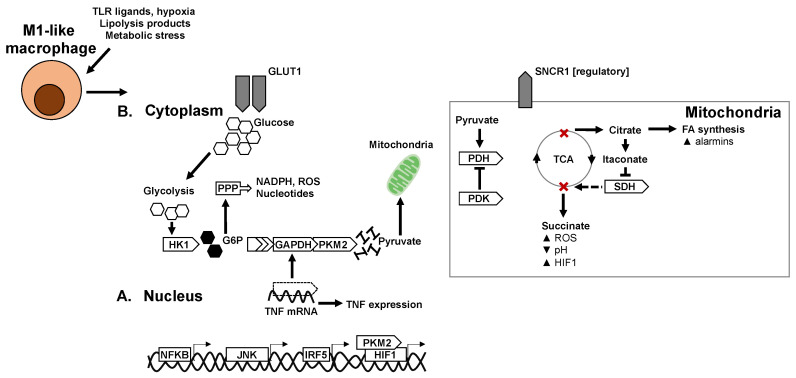
Transcriptional and metabolic adaptation of M1-like pro-inflammatory macrophages. Key transcription factors and enzymes that mediate pro-glycolytic and pro-inflammatory mechanisms in M1-like macrophages. (**A**) Transcriptional regulators of inflammation and macrophage metabolic adaptation. (**B**) Adaptation of metabolic pathways and enzymes in M1-like macrophages.

With ATMs being the central mediators of inflammation, much research has worked to decipher the mechanisms that control inflammatory polarisation and insulin resistance in the microenvironment. Multiple layers of regulation exist, from cell surface receptors to nuclear receptors, transcription factors and their co-regulators [17]. The multiple levels of complexity converge on the activation of two main inflammatory pathways: JNKs (c-Jun N-terminal kinases) and NFκB (nuclear factor kappa-light-chain-enhancer of activated B cells) [18,19,20]. These two pathways are active and exert their effects in both adipocytes and macrophages (Figure 1A).

Activation of these pathways leads to the production of pro-inflammatory cytokines, chemokines and chemoattractants that promote the recruitment of monocytes. Once recruited to AT, monocytes are exposed to the inflammatory milieu and differentiate in situ. These monocyte-macrophages may be phenotypically and functionally distinct from resident macrophages and are termed infiltrating macrophages [21]. Infiltrating macrophages amplify the local and systemic inflammatory responses that contribute to impaired insulin signalling [22].

### 4.1. JNK Signalling in Adipocyte Insulin Resistance

JNK is expressed in myeloid cells and in insulin target cells. JNK activation in myeloid cells results in the transcription of pro-inflammatory mediators. JNK responds to a number of stimuli, including inflammatory cytokines, free fatty acids, or intracellular signalling, such as the unfolded protein response [23,24]. Importantly, in insulin-target cells, JNK activation can inhibit the insulin signalling pathway through serine-threonine phosphorylation and inhibition of IRS-1. This results in inhibiting PI3K/protein kinase B (PKB) signalling downstream of IRS1. This role was deciphered with the use of JNK1-deficient mice, specific to adipose tissue [25,26,27].

### 4.2. NFκB Signalling in Adipocyte Insulin Resistance

With regards to NFκB, under physiological conditions, NFκB proteins are retained in the cytoplasm by inhibitors of κB (IκBs). Regulated proteasomal degradation of IκBs allows the nuclear translocation of NFκB; this leads to the transcription of canonical inflammatory mediators, such as interleukin (IL)-6, TNF, interferons and importantly the monocyte chemoattractant protein-1 (CCL2/MCP1), which is central to monocyte recruitment [28,29]. Adipocyte-specific IKKβ deletion and the proteins that mediate IκB degradation, results in a lack of responsiveness to fatty acids and a blunted inflammatory signature. Similarly, IKKβ overexpression in adipocytes decreases expression of anti-inflammatory molecules (leptin and adiponectin) [30].

### 4.3. Macrophage-Derived Cytokine Signalling and Adipose Tissue Inflammation

Innate immune cells, such as macrophages, mount their inflammatory responses following stimulation of pattern recognition receptors (PRRs). PRRs are composed of two main families: Toll-like and NOD-like receptors (TLRs and NLRs). These receptors span cell surface and intracellular membranes and are ligated by damage- or pathogen-associated molecular patterns (DAMPs or PAMPs). In the case of metabolic diseases, stimulation is by DAMPs generated by dysmetabolism, such as fatty acids, hyperglycaemia, cellular senescence or other stress signals [31].

Macrophages play a key role in amplifying inflammation in the adipose microenvironment. Once activated, secreted cytokines act both locally and peripherally to increase inflammation and insulin resistance [31]. Two main pathways are important actors of inflammation: the NLRP3 inflammasome and interferon signalling.

### 4.4. The NLRP3 Inflammasome: IL1B and IL18 Signalling

NLRP3 (gene encoding NALP3: NACHT, LRR and PYD domain-containing protein 3) is a NLR highly expressed in ATMs that forms a crown-like structure around senescent adipocytes upon diet-induced obesity [32]. NLRP3 and the adaptor protein ASC form a caspase-1 activating complex known as the NLRP3 inflammasome; the inflammasome complex is a sensor for metabolic homeostasis [33]. Activation of caspase-1 by this complex results in the maturation and release of IL1B and IL18; two potent inflammatory cytokines of the IL1 family. Mice deficient for NLRP3 are protected from diet-induced insulin resistance due to failure to form the inflammasome complex; similarly, therapeutic intervention for weight loss in obesity and T2D reduces expression of NLRP3 and its target cytokines [32].

IL1B is a key inflammasome product, a cytokine heavily involved in the development of T2D at multiple levels. Increased expression of IL1B is associated with insulin resistance as well as in the destruction of pancreatic beta cells in more advanced disease [34]. In AT, IL1B suppresses insulin signalling in adipocytes, exposure to IL1B results in decreased insulin stimulated glucose uptake and lipogenesis [35]. Glucose transporter (GLUT)-4 expression and translocation in adipocytes are also repressed [36,37].

IL1B exerts its effects by binding to its receptor and recruiting a coreceptor to form a heterodimer-receptor transmembrane complex. The cytoplasmic IL1R domain initiates intracellular signalling by recruiting the adaptor proteins MyD88 (myeloid primary response differentiation-88 protein) and IRAK (interleukin 1 associated kinase). Downstream of these adaptor proteins are the MAPK and NFκB signalling pathways, which are effectors of inflammation and insulin desensitisation.

IL18, another cytokine released by the inflammasome and member of the IL1 superfamily, is expressed in both immune and non-immune cells and is known to potentiate interferon signalling. Similar to IL1B, IL18 recruits the MyD88 adaptor protein and the inflammatory NFκB pathway. IL18 is released by AT and its circulating levels increase in obesity and T2D [38,39]. IL18 accelerates maturation of other immune cells, including T- and NK-cells, and enhances the production of other pro-inflammatory cytokines that exacerbate systemic inflammation in obesity and insulin resistance [40].

### 4.5. Macrophage-Derived IL6 and TNF Signalling

IL6 is an inflammatory cytokine involved in the development of insulin resistance [41]. IL6 expression characterises sepsis, mediating fever and the acute phase response. Mostly produced from macrophages, IL6 can also be released in small amounts by adipocytes; in AT, IL6 stimulates energy mobilisation and increases temperature. Upon interaction with its receptor, IL6 activates the JAK1-STAT3 (Janus kinase-signal transducer and activator of transcription) pathway [42]. In AT during obesity and insulin resistance, IL6 exposure impairs insulin signalling by interfering with IRS phosphorylation, thus increasing adipocyte lipolysis, which promotes hepatic gluconeogenesis and insulin resistance [43,44,45,46]. Although physiological and positive effects of IL6 action have also been reported (increased satiety, leptin release, IL4R expression in macrophages), the aberrant IL6 expression in diet-induced obesity results in chronic activation of its inflammatory axis.

Tumour necrosis factor (TNF) is an inflammatory cytokine expressed in two forms: a transmembrane form that mediates autocrine and paracrine signalling; and a soluble form that mediates endocrine signalling. The levels of both forms are increased in AT in obesity and insulin resistance with the main source being ATMs [47,48]. TNF interacts with the receptors TNFR1 and TNFR2; both receptors can also be cleaved creating soluble forms thought to neutralise and clear soluble TNF from circulation [49]. Ligation of TNFR1 mediates the majority of effects on adipocytes [50]. TNFR2 has been shown to contribute in a limited manner to altering adipocyte function, notably reducing GLUT4, and IRS-1 and -2 expression, inducing apoptosis or contributing to JNK activation [51,52,53]. Both TNFRs exert their effects through signal transduction and adaptor protein recruitment. The effects of TNF include inducing adipocyte and pre-adipocyte apoptosis, with pre-adipocytes being more sensitive [54]. Signalling downstream of TNFR also includes activation of NFκB and MAPK cascades and are leading candidates for mediating TNF-related metabolic dysregulation [55,56]. TNF action is associated with suppression of the insulin receptors IRS-1, -2 and GLUT4, through multiple mechanisms, including proteasomal degradation, impaired translocation and direct transcriptional repression [57,58,59].

## 5. Transcriptional Control of ATM Polarisation

### 5.1. Signal Transducers and Activators of Transcription: JAK-STAT Signalling

**Figure 2:**
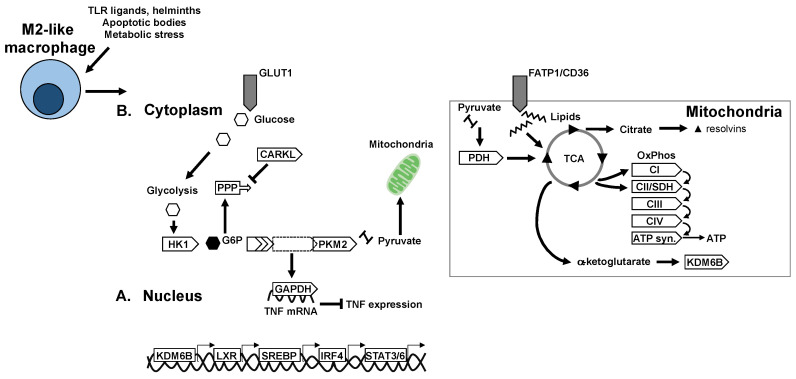
Transcriptional and metabolic adaptation of M2-like anti-inflammatory/pro-resolving macrophages. Key transcription factors and enzymes that mediate oxidative metabolism and immune-regulatory mechanisms in M2-like macrophages. (**A**) Transcriptional regulators of anti-inflammatory polarisation and macrophage metabolic adaptation. (**B**) Adaptation of metabolic pathways and enzymes in M2-like macrophages.

The JAK family of proteins interact with cytokine and growth factor receptors and mediates signalling to STAT proteins. STATs are a family of seven proteins that form part of the interferon signalling system, and have a cell- and tissue-specific distribution [60,61,62]. The JAK-STAT axis controls a large number of metabolic pathways in AT and is dysregulated in diet-induced obesity and insulin resistance [42,63]. STATs expressed in M2-like macrophages have been implicated in buffering pro-inflammatory polarisation in T2D (Figure 2A), whereas STATs in M1-like macrophages have not been directly linked to T2D.

STAT activity is regulated by cytokines and growth factors, which once signalled to membranes initiate JAK-mediated phosphorylation of STATs allowing their dimerization and nuclear translocation. STAT1 and STAT5 induce M1-like polarisation of macrophages, whereas STAT3 and STAT6 induce M2 macrophage polarisation. Whilst macrophage STAT1 has not been reported to play a role in obesity or insulin resistance; in vitro and ex vivo studies demonstrated that STAT1 is activated in response to high glucose where a large epigenetic component is attributed to its function [64]. Similarly, no conclusive studies have linked STAT5 to T2D pathogenesis to our knowledge.

M2 polarising STATs are more closely associated to T2D. STAT3 is a downstream target of metformin. Metformin inhibits differentiation of monocytes to macrophages through AMPK-mediated inhibition of STAT3, which also resulted in decreased monocyte infiltration into atherosclerotic plaques and an ameliorated outcome in mice [65]. In other studies, a model of myeloid-specific deficiency of JAK2, reduced phosphorylation of STAT3 and led to a less inflammatory and healthier visceral AT phenotype upon diet-induced obesity and insulin resistance [66]. Mice deficient for STAT6 are more prone to diet-induced obesity and increase in oxidative stress and inflammation in AT, increasing susceptibility to insulin resistance and T2D [67].

### 5.2. Type-1 Interferon Signalling and Transcription Control

The type-1 interferons (IFN) are a large family of proteins that regulate the multiple components of the immune system and were initially characterised as the host response to viral infection. IFNs signal danger to cells in proximity causing them to heighten their anti-viral defences [68]. However, over the years, several other roles have been attributed to IFNs, mainly in autoimmune and metabolic diseases. In macrophages, and other cells of the myeloid system, type-1 IFNs (IFNα, β, κ and ω) are expressed [69]. They are stimulated downstream of TLR-ligation, canonically by bacterial or viral stimuli, whereas the stimuli in metabolic diseases remain to be elucidated. Macrophage type-1 IFN expression is tightly regulated by two transcription factors, the IFN regulatory factors (IRF)-4 and -5.

Many studies have solidified the roles of these two transcription factors in the pathogenesis of T2D [70]. M1-like polarisation of macrophages is promoted by IRF5, whereas pro-resolution, M2-like polarisation is driven by IRF4 [71]. In ATMs, IRF5 is instrumental in mediating metabolic inflammation, leading to maladaptive AT expansion and the development of insulin resistance [14]. Mice with a myeloid-deficiency of IRF5 have improved metabolic homeostasis upon high-fat feeding, lower AT inflammation and redistribution of AT from the visceral depot towards the subcutaneous depot. This phenomenon is mediated by alternatively activated macrophages that restrict adipocyte growth and promote a hyperplasic and metabolically protective response upon diet-induced obesity. Interestingly this phenotype is comparable to that of TLR4-deficient mice, indicating that the TLR4–IRF5 axis of macrophage polarisation may be conserved in metabolic inflammation [72]. With regards to IRF4, deficiency in myeloid cells results in mice with exuberant AT inflammation and decreased insulin sensitivity when compared to IRF4-competent mice upon diet-induced obesity, despite no differences in weight gain [73]. Interestingly, IRF4 is also expressed in adipocytes, where it is required for lipolytic programming; mice lacking IRF4 in adipocytes are predisposed to increased weight gain and adiposity and deficient lipolysis [74].

## 6. Metabolic Mechanisms of Macrophage Polarisation

Macrophage cellular metabolism, or bioenergetic adaptation, also strongly influences effector functions. Canonically, pro-inflammatory polarisation is associated with glycolytic metabolism whereas anti-inflammatory or pro-resolution polarisation is associated with oxidative or mitochondrial metabolism (Figure 1B and Figure 2B). These metabolic pathways and the associated functions have been established in recent years with the application of model systems (such as bone marrow derived macrophages) treated with model immunogens or cytokines. The exact stressors in a diabetogenic context and the specific metabolic adaptations of tissue macrophages in situ remains to be fully deciphered. The particularity of diabetogenic conditions is the systemic abundance of metabolic substrates in hyperglycaemia and dyslipidaemia. Moreover, AT is particularly rich in lipids and lipolysis products at the onset of insulin resistance and thus ATMs are expected to have particular adaptations to this microenvironment and metabolic milieu (Table 1).

### 6.1. Glycolysis in Macrophage Polarisation

Typically, in predominantly glycolytic macrophages, that are pro-inflammatory, the rapid influx of glucose is supported by upregulation and plasma membrane localisation of GLUT1 [75]. However, in some primary macrophage subsets, the increased uptake and metabolism of glucose has been shown to be dissociable from inflammation, notably in lipid-rich microenvironments such as in atherosclerotic plaques [76]. GLUT1 is indeed expressed in AT, and specifically in ATM, that forms crown-like structures over the course of diet-induced obesity, however a specific role for GLUT1 in ATM function or the development of insulin resistance remains to be validated (Figure 1).

Components of the glycolytic pathway have adjunct functions in macrophages, some components interact with transcriptional and inflammatory machinery to influence polarisation. Hexokinase (HK)-1 is a key enzyme that phosphorylates glucose to produce glucose-6-phosphate as the first step of glycolysis. In macrophages, HK1 promotes NLRP3 inflammasome formation and supports IL1B secretion, in collaboration with the mammalian target of rapamycin complex (mTORC)-1 [77].

The hypoxia sensor hypoxia-inducible factor (HIF)-1-α is an important link between glycolytic programming and inflammation. HIF1α is stabilised in macrophages by succinate, a metabolite of the tricarboxylic acid (TCA) cycle that accumulates with increasing glycolytic flux [78]. The stabilisation of HIF1α allows its transcriptional activity to take place, inducing expression of GLUT1 as well as the expression of inflammatory mediators such as IL1B, with the pyruvate kinase isoenzyme (PKM)-2 as a coregulator. PKM2 canonically catalyses the last step of glycolysis, being responsible for net ATP and pyruvate production. In M1 macrophages, it also acts as a transcriptional coregulator [79,80]. The HIF1α pathway has itself been validated to play a role in metabolic diseases, where HIF1α deficient mice are protected against diet-induced obesity and insulin resistance.

Another glycolytic enzyme that influences inflammation is glyceraldehyde 3-phosphate dehydrogenase (GAPDH). GAPDH binds TNF at the mRNA level and post-transcriptionally represses its expression in monocytes [81]. When glycolysis is up-regulated, GAPDH is recruited to carry out its metabolic function, and this releases its break on TNF mRNA, allowing maturation and release of the cytokine in its active form.

The carbohydrate kinase-like (CARKL) protein is downregulated in classical macrophages. Downregulation channels the flux of glycolytic intermediates through the pentose phosphate pathway (PPP). Increased PPP activity generates substrates for protein and nucleotide synthesis, as well as the production of NADPH and NADPH oxidase-mediated ROS [82]. These activities support cytokine synthesis and generation of products necessary for bacterial killing.

As well as metabolic enzymes and metabolites that interact with inflammatory machinery, inflammatory transcription factors, namely IRF5, have been implicated in the control of cellular metabolism. Two separate mechanisms have been demonstrated for the role of IRF5 in cellular metabolism, the impairment of efferocytosis and the promotion of glycolysis. Seneviratne et al. demonstrated that IRF5 supports foam cell formation in atherosclerosis by inhibiting necrotic core formation, this would lead to an increase and possible overload of substrates in lipid-laden macrophages [83]. Conversely, genetic risk-variants that increase IRF5 expression are associated with increased glycolysis in bone-marrow derived macrophages [84]. Such mechanisms clearly work in parallel to IRF5′s main function as a transcription factor promoting M1 polarisation.

Much less frequently, glycolysis has also been reported in alternatively activated macrophages. Precisely, macrophage stimulation with IL4 and MCSF leads to activation of mTORC2 and the upregulation of glycolysis through IRF4 [85]. However, a subtle difference in the glycolytic programming of alternatively activated macrophages is that glycolysis products are directed towards mitochondria rather than towards the PPP. This supports the TCA cycle and mitochondrial oxidative phosphorylation (OXPHOS); accordingly, other studies have shown that 2-deoxyglucose mediated inhibition of glycolysis also attenuates mitochondrial metabolism and M2 marker expression in response to IL4 [86].

### 6.2. Mitochondria and Mitochondrial Respiration in Macrophage Polarisation

Mitochondria have numerous functions beyond their roles in oxidative metabolism, such as dictating levels of oxidative stress, calcium signalling, apoptosis, and inflammation, as well as cellular and systemic metabolism. Altered mitochondrial function and reduced mitochondrial DNA have been reported in murine models of genetic obesity [87]; with decreases in mitochondrial activity also reported in human AT from obese individuals [88]. A general mechanism by which mitochondria contribute to inflammation is interaction with redox sensitive pathways in the cell. Mitochondrial dysfunction leads to aggravated inflammation through activation of redox-sensitive inflammatory mechanisms, such as NFκB or the NLRP3 inflammasome [89,90]. Mitochondrial dysfunction is also associated with accumulation of ectopic or excess lipids, interfering with insulin signalling and leading to adipocyte hypertrophy and hypoxia, respectively [91].

In terms of metabolism, mitochondria metabolise lipids as well as glycolysis end-products. Increased lipid uptake and oxidation characterise alternatively activated macrophages following IL4 stimulation [92]. Indeed, inhibition of fatty acid oxidation (FAO) or OXPHOS attenuates expression of M2 markers [93,94]. In the case of IL4 stimulation, metabolic reprogramming of M2 polarisation is mediated by STAT6 and the peroxisome proliferator-activated receptor gamma (PPARγ), coactivator 1 beta (PGC1B) [95]. The substrates for FAO are exogenous lipids taken up by CD36 or are synthesised by lipogenic machinery of the cell. An additional important step for metabolising lipid imported into the cell by CD36 is their lipolysis by the lysosomal acid lipase (LAL). LAL deficiency or treatment by orlistat, a lipolysis inhibitor, decreases macrophage oxidative respiration and impairs alternative polarisation [96].

Fatty acid transporters (FATP1) and fatty acid binding proteins (FABP4/5) also influence macrophage polarisation [96,97]. Macrophage-deficiency of FATP1 biases cellular respiration towards glycolysis and primes polarisation towards a pro-inflammatory phenotype, which is reflected in vivo by an aggravated metabolic phenotype in diet-induced obesity. Interestingly, the deletion of FABP4/5 in macrophages results in a metabolically protective phenotype. This indicates that FABPs may contribute more to signal transduction from a dysmetabolic environment than to substrate provision for oxidative cellular respiration.

Mitochondrial enzymes themselves contribute to regulation of cellular metabolism and macrophage fate (Figure 2). Carnitine palmitoyl transferase (CPT)-1 is an enzyme essential for movement of substrates from the cytoplasm to the mitochondria for their metabolism. Overexpression of CPT1 increases FAO while decreasing triglyceride content and production of pro-inflammatory cytokines [98]. Alternatively, the loss of CPT2 in macrophages results in decreased FAO with no functional consequence on the IL4-stimulated alternative activation phenotype [94,99]. Similarly, etomoxir inhibition of FAO does not affect alternative activation of macrophages. Taken together these data indicate that CPT1 has specific properties that mediate anti-inflammatory/alternative polarisation, which may only be fuelled or potentiated by the other components of the CPT-FAO axis. Alternatively, FAO and CPT2 may play more significant roles in regulating redox status, or in tempering and resolving pro-inflammatory polarisation.

### 6.3. TCA Cycle and Intermediates in Macrophage Polarisation

The TCA cycle plays a central role in both pro- and anti-inflammatory polarization. Under classical activation, the TCA cycle is interrupted at two steps that result in decreased alpha-ketoglutarate (αKG) production and accumulation of succinate and citrate. Citrate is exported to the cytoplasm as a substrate for lipogenesis, where products are alarmins and pro-inflammatory mediators such as prostaglandin E2 and nitrous oxide [100]. Citrate can also be converted to itaconate through the enzyme immune responsive gene (IRG)-1. Itaconate is a metabolite that inhibits SDH and allows the accumulation of succinate [101]. The accumulation of succinate stabilizes HIF1α, which is an integral part of glycolytic and inflammatory programming. Moreover, in the presence of oxygen, succinate oxidation also provides a source of mitochondrial ROS necessary for anti-bacterial activity [102]. More recently another mechanism described autocrine as well as paracrine roles of succinate signalling, where succinate can be secreted by the succinate receptor (SUCNR)-1. In this context, a polarized macrophage can act on macrophages in the vicinity through succinate as a metabokine [103,104]. Interestingly, extracellular succinate plays a different role to intracellular succinate, as mice with SCNR1 deficiency in macrophages, develop a more aggravated metabolic response to high-fat feeding with a hyperinflammatory profile in AT [104].

αKG is a ketone derivative and intermediate of the TCA cycle, produced by oxidative decarboxylation of isocitrate or by oxidative deamination of glutamate. Several recent studies have revealed important roles of αKG in controlling macrophage polarization through metabolic and epigenetic mechanisms. A high αKG to succinate ratio promotes alternative (M2) activation of macrophages by engaging FAO and acting as a co-factor for the lysine demethylase (KDM)-6B [105]. High levels of αKG promote tolerance and resolution of inflammation after M1 polarisation.

Just upstream of the TCA cycle, pyruvate dehydrogenase kinase (PDK) hinders the transport of carbon substrates to the TCA cycle by repressing pyruvate dehydrogenase (PDH). This biases towards glycolysis and thus inflammation. Indeed, pharmacologic inhibitors of PDK mitigate inflammatory polarization. In vivo myeloid-deficiency of PDK improves the metabolic phenotype of mice upon a high-fat diet with a decrease in AT inflammation and decreased ATM accumulation [106,107].

## 7. Nuclear Receptors and Transcriptional Control of Macrophage Metabolism

The main cellular pathways in lipogenesis and lipid metabolism are put in motion by the nuclear receptors PPARγ and PPARβ/δ. Deletion of either protein in macrophages inhibits alternative polarisation in response to IL4 [108]. In vivo and upon high fat feeding, the deletion of PPARγ or PPARβ/δ similarly results in mice with an exacerbated metabolic phenotype and increased adipose tissue inflammation [109].

Liver X receptors (LXRs) are also expressed in macrophages; they act as liposensors, regulating intracellular cholesterol content and controlling the expression of efflux transporters ABCA1 and ABCG1, and apolipoproteins APOE and APOC [110,111]. In vitro, LXR ligands inhibit the expression of inflammatory cytokines and promote the expression of arginase (ARG)-2, an enzyme characteristic of alternative polarisation that potentiates the anti-inflammatory response [112].

Sterol regulatory element binding proteins (SREBPs) are the master regulators of lipogenesis, especially de novo lipogenesis, promoting transcription of key enzymes for fatty acid synthesis (e.g., FASN, ACC, SCD1) [113]. However, their roles in immune cells are emerging, and have so far proven to be diverse. SREBP1 enhances expression of NLRP1A, a component of the NLRP3 inflammasome that culminates in the release of IL1B, as well as being necessary for signalling downstream of TLR4 [114,115]. SREBP1a does however have a dual role where it synthesises unsaturated anti-inflammatory fatty acids, or resolvin lipids. Despite its role in the expression of genes that lead to completion of inflammasome assembly, the loss of resolvins has a more important functional effect, where the response to TLR stimulation in vitro is of exaggerated inflammation [116]. In the context of insulin resistance and T2D, deficiency of the SREBP target gene fatty acid synthase (FASN) in macrophages leads to a hyperinflammatory and metabolically perturbed phenotype upon diet-induced obesity. This occurs as FASN plays such a crucial role in producing cellular lipids, including lipids that form the basis of membranes and their downstream signalling. Thus, the lack of FASN resulted in the production of disordered and functionally compromised membranes that aggravated macrophage responses [117]. Taken together, these data indicate that lipid metabolism in macrophages is complex and is of considerable functional importance. This field of work is still in its infancy in the domain of T2D and obesity, and could benefit from research into foam cell biology from the fields of atherosclerosis and cardiovascular disease.

## 8. Perspectives and Concluding Remarks

Here we have reviewed the roles and regulation of inflammation in the development of insulin resistance and in maladaptive AT function in obesity and T2D. Whilst transcriptional and molecular mechanisms linking inflammation to insulin resistance in AT have been extensively studied, several questions remain unanswered.

The factors that initiate ATM activation remain largely unknown in diabetogenesis. Several cues have been implicated, and are attractive candidates, such as fatty acids, hyperglycaemia or adipocyte death. However, these factors have all also been reported to be aggravated by, or to be downstream of inflammatory polarisation. Thus, defining the metabolic immunogens, or danger signals, that induce sterile inflammation in insulin resistance and T2D is an active area of research.

As we have largely focused on in this review, macrophages undergo extensive bioenergetic and metabolic adaptation to carry out their effector functions. Much progress has been made to determine the fundamental metabolic mechanisms that fuel macrophage polarisation; these early studies have largely been carried out in model systems with canonical PRR ligands. Future work could characterise the metabolic requirements of macrophages that populate specific tissue niches as well as macrophage activation in specific disease contexts.

Lastly, the translation aspects of fundamental research must be addressed. To date, a major challenge has been in the specific targeting of macrophages or other innate immune cells without affecting other cell types. Advances have been made in these domains, such as conjugation of bioactive molecules to antibodies targeting phenotypic cell surface receptors [118].

## Figures and Tables

**Table 1:** Major adaptations in ATM polarisation states. When exposed to specific stimuli, macrophages undergo terminal differentiation into M1-like, M2-like or intermediate polarisation states. These states imply the activation of several transcriptional and metabolic pathways.

	Insulin Resistant State	Insulin Sensitive State
ATM polarisation	M1-like	M2-Like
Canonnical stimuli	Bacterial/viral stimuli (TLR/NLR ligands)	Helminths (TLR, lectin receptors)
Stimuli in T2D	Inflammatory cytokines/chemokines Free fatty acids, Hypoxia TLR/NLR ligands	Regulatory/Anti-inflammatory cytokines/chemokines Fatty acids, apoptotic bodies, CD36/FATP1 ligation Metformin, hormone signalling (leptin, adiponectin)
Pathway/TFs	JNK, NFKB, NLRP3, IRF5, HIF1	STAT3/6, IRF4, KDM6B, LXR, SREBPs
Cytokines/Chemokines	IL1B, IL18, IL6, TNF, IFNα, β, κ and ω	IL10, TGF, CCL1, IL1Ra *succinate
Glycolysis and PPP adaptation	GAPDH releases nuclear break on TNF mRNA ↘ CARKL, ↗ PPP ↗ PPP provides substrates for protein and nucleotide synthesis ↗ GLUT1 ↗ glucose uptake ↗ HK1 supports NLRP3 inflammasome ↗ Succinate stabilises HIF1 PKM2 is coregulator for HIF1 ↗ IRF5 ↗ AKT2 glycolysis	GAPDH reatined in nucleas to bind TNF ↗ CARKL ↘ PPP ↗ Pyruvate towards mitochondria mTORC sustains glycolysis
Mitochondria and oxidative metabolism	↘ Oxidative capacity ↗ Citrate conversion to itaconate by ↗ IRG1 ↗ Itaconate inhibits SDH ↘ SDH ↗ succinate ↗ Succinate ↘ pH ↗HIF1 PDK inhibition of PDH ↘ Pyruvate flux to mitochondria Citrate subtrate for alarmin lipids Succinate oxidation ↗ bactericidal ROS	↘ IRF5 ↗ efferocytosis ↗ CD36/FATP1 ↗ Lipid uptake ↗ PPARs/STAT6 ↗ Lipogenesis ↗ lipid substrates for mitochondrial respiration and FAO ↗ CPT1 ↗ substrates to mitochondria TCA cycle is intact OxPhos maintains redox balance Secreted succinate has a regulatory effect in microenvironment ↗ αKG cofactor for KDM6b ↗ KDM6b ↗ anti-inflammatory programming

* succinate is a metabokine, metabolite signaling molecule.

## Abbreviations

T2D	Type-2 diabetes
AT	Adipose tissue
ATM	Adipose tissue macrophages
SVF	Stromal vascular fraction
TNF	Tumour necrosis factor
IRS	Insulin receptor substrate
PI3K	Phosphatidylinositol 3-kinase
MAPK	Mitogen-activated protein kinase
JNK	c-Jun N-terminal kinase
NFκB	Nuclear factor kappa-light-chain-enhancer of activated B cells
PKB	Protein kinase B
IκB	Inhibitors of κB
IL6	Interleukin 6
CCL2/MCP1	Chemokine (C-C motif) ligang 2/Monocyte chemoattractant protein 1
IKKβ	Inhibitor of nuclear factor kappa-B kinase subunit beta
PPR	Pattern recognition receptors
TLR	Toll-like receptors
NLR	NOD-like receptors
DAMP	Damage-associated molecular pattern
PAMP	Pathogen-associated molecular pattern
NLRP3	Gene encoding NACHT, LRR and PYD domains-containing protein 3
ASC	Apoptosis-associated speck-like protein containing a CARD
IL1B	Interleukin 1 beta
IL18	Interleukin 18
IL1R	Interleukin 1 receptor
MyD88	Myeloid primary response differentiation-88 protein
IRAK	Interleukin 1 associated kinase
JAK	Janus kinase
STAT	Signal transducer and activator of transcription
IL4R	Interleukin 4 receptor
TNFR	TNF receptor
AMPK	AMP-activated protein kinase
IFN	Interferon
IRF	Interferon regulatory factor
GLUT	Glucose transporter
HK	Hexokinase
mTORC1	Mammalian target of rapamycin complex
HIF1	Hypoxia inducible factor-1
TCA	tricarboxylic acid cycle
GAPDH	Glyceraldehyde 3-phosphate dehydrogenase
CARKL	Carbohydrate kinase-like
PPP	Pentose phosphate pathway
NADPH	Nicotinamide adenine dinucleotide phosphate
ROS	Reactive oxygen species
MCSF	Macrophage colony-stimulating factor
OXPHOS	oxidative phosphorylation
FAO	Fatty acid oxidation
IL4	Interleukin 4
LAL	Lysosomal acid lipase
FATP1	Fatty acid transporter 1
FABP	Fatty acid binding protein
CPT	Carnitine palmitoyl transferase
αKG	Alpha-ketoglutarate
SUCNR1	Succinate receptor
PDH	Pyruvate dehydrogenase
PDK	Pyruvate dehydrogenase kinase
PPAR	Peroxisome proliferator-activated receptor
ABCA1	ATP-binding cassette transporter sub-family A member 1
ABCG1	ATP-binding cassette transporter sub-family G member 1
APOE	Apolipoprotein E
APOC	Apolipoprotein C
ARG2	Arginase-2
SREBP	Sterol regulatory element binding protein
NLRP1A	Gene encoding NACHT, LRR and PYD domains-containing protein 1A
FASN	Fatty acid synthase
